# Mediation of endothelial activation and stress index in the association between vitamin B_6_ turnover rate and diabetic retinopathy: an analysis of the National Health and Nutrition Examination Survey

**DOI:** 10.3389/fnut.2024.1490340

**Published:** 2025-01-14

**Authors:** Jie Yin, Juan Chen, Yuanyuan Chen

**Affiliations:** Department of Ophthalmology, The Affiliated Hospital of Hangzhou Normal University, Hangzhou, China

**Keywords:** mediating effect, DR, 4-PA/PLP, EASIX, distribution-of-product method

## Abstract

**Aim:**

This study aimed to explore the association between the ratio of 4-pyridoxine (4-PA) to pyridoxal 5′-phosphate (PLP) (4-PA/PLP) and diabetic retinopathy (DR) and further assess the mediating effect of Endothelial Activation and Stress Index (EASIX) on the association between 4-PA/PLP and DR.

**Methods:**

In this cross-sectional study, 1,698 patients with diabetes from the National Health and Nutrition Examination Survey were included. According to the median, 4-PA/PLP was categorized into a high-level group (≥0.89) and a low-level group (<0.89). As the EASIX had a skewed distribution, it was log-transformed before analysis. Weighted logistic regression models were used to investigate the association of 4-PA/PLP with EASIX and DR and the mediating effect of EASIX on the association between 4-PA/PLP and DR. The distribution-of-product method was adopted to assess the mediating effect. Subgroup analysis was performed based on the age and duration of diabetes.

**Results:**

A total of 362 diabetic patients were classified as having DR. After adjusting for all covariates, a higher level of 4-PA/PLP was associated with an increased level of log EASIX [odds ratio (OR) = 1.56, 95% confidence interval (CI): 1.06–2.30]. A higher ratio of 4-PA/PLP was associated with increased odds of DR compared to the reference group with lower levels of 4-PA/PLP (OR = 1.94, 95%CI: 1.40–2.67). In addition, we found that log EASIX may play a mediating role in the 4-PA/PLP and DR, with a 95% CI of distribution of product of 0.31 (95% CI: 0.02–0.67). The proportion of mediation was 69.06%. The mediating effect of log EASIX was also observed in individuals with diabetes who were aged≥60 years (proportion of mediation: 50.63%) or had a duration of diabetes ≥10 years (proportion of mediation: 71.83%).

**Conclusion:**

This study found a positive association between high levels of 4-PA/PLP and an increased risk of DR, with the relationship being partially mediated by log EASIX.

## Introduction

Diabetic retinopathy (DR) is one of the most common and specific microvascular complications of diabetes (1). According to the report of a systematic review and meta-analysis, the global population with DR was estimated to be 103.12 million in 2020 and is projected to increase to 160.50 million in 2045 (2). DR not only significantly contributes to the development of blindness and visual impairment but also is associated with an increased risk of all-cause mortality (3). DR has imposed a significant disease burden worldwide (2).

Endothelial dysfunction is an important pathological mechanism for the occurrence and development of DR (4, 5). However, the accuracy and reproducibility of assessing endothelial function, specifically flow-mediated dilation, are susceptible to operator variability, and there is no universally recognized biomarker for evaluating endothelial function. Recently, a novel Endothelial Activation and Stress Index (EASIX) based on biochemical markers including lactate dehydrogenase (LDH), creatinine, and platelets has been proposed and utilized for investigating the prognostic implications in patients with severe diseases and cancer (6, 7). However, there is currently a lack of research exploring the association between the EASIX and DR.

Vitamin B_6_ is a crucial micronutrient for the human body, and its anti-inflammatory properties have been progressively elucidated (8). Recent studies have indicated that reduced vitamin B_6_ intake may be linked to an elevated risk of developing DR and an increased mortality rate in patients with DR (9, 10), potentially due to the suppression of superoxide production in microvascular endothelial cells and the inflammatory response (10, 11). It is well known that dietary intake may not accurately reflect the body’s vitamin B_6_ levels and does not reflect the catabolic status of vitamin B_6_ catabolism status. Several studies have found that vitamin B_6_ catabolism, or vitamin B_6_ turnover, was strongly correlated with the risk of mortality (12, 13) and cardiovascular disease (CVD) (14) in individuals with diabetes. This association is reflected by the ratio of 4-pyridoxine (4-PA) to pyridoxal 5′-phosphate (PLP) (4-PA/PLP), a blood marker that is linked to vitamin B_6_ levels. This ratio integrates changes in vitamin B_6_ metabolism mediated by inflammation and serves as a functional indicator of inflammatory status and dysregulation in vitamin B_6_ metabolism (14). Chronic inflammation plays a crucial role in the pathogenesis of DR associated with endothelial dysfunction (4, 5). However, few studies have investigated the correlation between the 4-PA/PLP ratio and DR, as well as whether this ratio influences DR risk through its impact on endothelial function.

In light of this background, our study aimed to explore the association between 4-PA/PLP and DR and further assess the mediating effect of EASIX on this association. It is hoped that this study could contribute to the early identification of high-risk populations and provide some references for DR management.

## Methods

### Data sources

In this cross-sectional study, all data were obtained from the National Health and Nutrition Examination Survey (NHANES) database. The NHANES is a complex, multistage, and probabilistic sampling design survey, which combines interview information with physical examination data (15). All NHANES participants provided written consent to participate in the survey, and their data collection received approval from the National Center for Health Statistics Research Ethics Review Board.

### Study eligibility criteria

**Figure 1:**
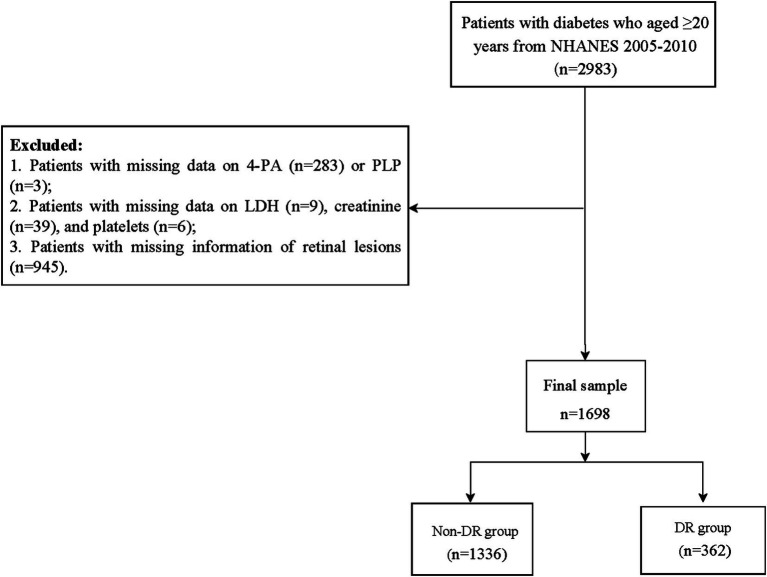
Flowchart of the participants screening. NHANES, National Health and Nutrition Examination Survey; 4-PA, 4-pyridoxine; PLP, pyridoxal 5′-phosphate; LDH, lactate dehydrogenase; DR, diabetic retinopathy.

Initially, our study included 2,983 patients with diabetes who were aged ≥20 years from the NHANES 2005–2010. Of those, 1,285 patients with diabetes were excluded from this study due to the following reasons: (1) patients with missing data on 4-PA (*n* = 283) or PLP (*n* = 3); (2) patients with missing data on LDH (*n* = 9), creatinine (*n* = 39), and platelets (*n* = 6); and (3) patients with missing information of retinal lesions (*n* = 945). Finally, 1,698 patients with diabetes were included in our final analyses and categorized into two groups based on DR status [DR group (*n* = 362) and non-DR group (*n* = 1,336)] (Figure 1).

### Data collection

#### Assessment of diabetes and DR

Diabetes was defined based on self-reported diagnosis, use of hypoglycemic medication, or the following criteria: glycohemoglobin A1c (HbA1c) ≥6.5%, fasting glucose ≥126 mg/dL, and 2-h plasma glucose ≥200 mg/dL during the oral glucose tolerance test (OGTT) (16). The assessment for DR was based on individuals who responded “Yes” to the question “Has a doctor ever told you that diabetes has affected your eyes or that you have retinopathy?”.

#### Measurement of 4-PA/PLP and EASIX

For NHANES participants, the serum 4-PA and PLP concentrations were determined using high-performance liquid chromatography. More methodological details can be found in the laboratory procedure manual provided by the NHANES: https://wwwn.cdc.gov/Nchs/Nhanes/2009-2010/VIT_B6_F.htm. 4-PA/PLP was calculated as 4-PA÷PLP concentration. Based on the median, 4-PA/PLP was categorized into a high-level group (≥0.89) and a low-level group (<0.89).

The EASIX score was calculated using the formula: LDH (U/L) × creatinine (mg/dL) /platelet count (10^9^/L). As the EASIX had a skewed distribution, it was log-transformed before analysis. Log EASIX was divided into two groups: high (≥ − 0.73) and low (<−0.73).

#### Covariates

Patient information collected included age, gender, ethnicity, education level, family poverty-to-income ratio (PIR), smoking status, physical activity, duration of diabetes, hypertension, dyslipidemia, CVD, cancer, body mass index (BMI, kg/m^2^), energy (kcal), vitamin B_6_ (mg), vitamin B_12_ (mcg), folate (mcg), and C-reactive protein (CRP, mg/dL). A history of hypertension was defined as a self-reported physician diagnosis, systolic blood pressure (SBP) ≥130 mmHg, diastolic blood pressure (DBP) ≥80 mmHg, or use of antihypertensive medication. The duration of diabetes was calculated by subtracting the age at onset from the current age. In the NHANES database for 2005–2006, no information regarding the supplement usage of vitamin B6, vitamin B12, and folate was available. In the NHANES 2007–2010, the prevalence of missing data for vitamin B6 supplements was approximately 75.46%, while for vitamin B12 supplements, it was approximately 74.27%, and for folate supplements, it was approximately 75.69%. Given that there are too many missing or no data on vitamin B6, vitamin B12, and folate supplements in the NHANES database, the data collected for this study solely relied on dietary intake. The data on vitamin B6, vitamin B12, and folate were estimated by calculating the daily diet intake obtained from the NHANES Dietary Data section.

### Statistical analysis

Considering the complex survey design in the NHANES, all analyses applied the appropriate sampling weights for NHANES data. Weighted variables such as SDMVPSU, SDMVSTRA, and WTMEC2YR were used in this study. Categorical variables were expressed as the number of cases and composition ratio [*n* (%)] and were statistically analyzed between groups using the chi-square test. Continuous variables were expressed as mean ± standard error (SE) and were analyzed between groups using a weighted *t-*test. Missing variables were interpolated using the multiple imputation method, and sensitivity analysis is shown in Supplementary Table S1.

Weighted logistic regression models were used to investigate the association of 4-PA/PLP with EASIX and DR and the mediating effect of EASIX on the association between 4-PA/PLP and DR. The distribution-of-product method was adopted to assess the mediating effect, with the 95% confidence interval (CI) for the product distribution calculated using the “RMediation” software package. A mediation effect is considered significant when the CI does not include 0. The proportion of mediation was calculated based on both indirect and total effects. In addition, subgroup analysis was performed to test whether the mediating effect of EASIX could be altered by age and duration of diabetes. The odds ratio (OR) with a corresponding 95% CI was calculated. *p* < 0.05 was considered statistically significant.

## Results

### General characteristics of study participants

A total of 1,698 patients with diabetes were included in our study. Table 1 shows the general characteristics of all included participants, with 48.27% of the participants being male. The average age of patients with diabetes was 59.39 (0.52) years. The mean BMI was 32.77 (0.26) kg/m^2^. Among the 1,698 patients, 362 patients with diabetes were classified as having DR. In general, participants with DR were more likely to have a lower proportion of PIR, longer duration of diabetes, higher proportion of CVD, and higher levels of creatinine, LDH, and EASIX than those without DR.

**Table 1:** General characteristics of study participants.

Variables	Total (*n* = 1,698)	Non-DR group (*n* = 1,336)	DR group (*n* = 362)	Statistics	*P*
Age, years, mean (S.E)	59.39 (0.52)	59.41 (0.57)	59.31 (0.98)	*t* = 0.09	0.927
Sex, *n* (%)				χ^2^ = 1.351	0.245
Male	851 (48.27)	677 (49.20)	174 (44.72)		
Female	847 (51.73)	659 (50.80)	188 (55.28)		
Ethnicity, *n* (%)				χ^2^ = 2.043	0.360
White	668 (62.44)	539 (63.15)	129 (59.72)		
Black	463 (16.89)	356 (16.24)	107 (19.36)		
Others	567 (20.67)	441 (20.61)	126 (20.93)		
Education level, n (%)				χ^2^ = 1.221	0.543
Below high school	699 (29.60)	544 (28.83)	155 (32.58)		
High school	378 (24.22)	302 (24.35)	76 (23.71)		
Above high school	621 (46.18)	490 (46.82)	131 (43.71)		
PIR, *n* (%)				χ^2^ = 13.991	<0.001
<1	352 (14.01)	258 (12.55)	94 (19.57)		
≥1	1,346 (85.99)	1,078 (87.45)	268 (80.43)		
Smoking status, *n* (%)				χ^2^ = 0.192	0.661
No	1,422 (83.88)	1,119 (83.65)	303 (84.76)		
Yes	276 (16.12)	217 (16.35)	59 (15.24)		
Physical activity, *n* (%)				χ^2^ = 5.006	0.082
<750 MET·min/week	952 (51.18)	741 (50.11)	211 (55.26)		
≥750 MET·min/week	513 (33.70)	424 (35.29)	89 (27.61)		
Unknown	233 (15.13)	171 (14.60)	62 (17.13)		
Duration of diabetes, years, mean (S.E)	10.84 (0.30)	9.78 (0.31)	14.88 (0.69)	*t* = −6.73	<0.001
Duration of diabetes, years, *n* (%)				χ^2^ = 34.018	<0.001
<10	910 (55.75)	790 (60.68)	120 (36.86)		
≥10	788 (44.25)	546 (39.32)	242 (63.14)		
Hypertension, *n* (%)				χ^2^ = 0.962	0.327
No	201 (14.02)	165 (14.52)	36 (12.08)		
Yes	1,497 (85.98)	1,171 (85.48)	326 (87.92)		
Dyslipidemia, *n* (%)				χ^2^ = 2.000	0.157
No	180 (12.08)	155 (12.86)	25 (9.09)		
Yes	1,518 (87.92)	1,181 (87.14)	337 (90.91)		
CVD, *n* (%)				χ^2^ = 8.064	0.005
No	863 (53.41)	717 (55.91)	146 (43.86)		
Yes	835 (46.59)	619 (44.09)	216 (56.14)		
Cancer, *n* (%)				χ^2^ = 0.361	0.548
No	1,460 (85.59)	1,138 (85.20)	322 (87.06)		
Yes	238 (14.41)	198 (14.80)	40 (12.94)		
BMI, kg/m^2^, mean (S.E)	32.77 (0.26)	32.60 (0.27)	33.43 (0.64)	*t* = −1.19	0.242
BMI, kg/m^2^, n (%)				χ^2^ = 0.255	0.880
<25	230 (13.36)	184 (13.59)	46 (12.51)		
25 ~ 30	476 (25.78)	375 (25.94)	101 (25.17)		
≥30	992 (60.85)	777 (60.47)	215 (62.32)		
Energy (kcal), mean (S.E)	1870.54 (29.11)	1882.68 (33.79)	1824.04 (49.96)	*t* = 0.98	0.330
Vitamin B_6_ (mg), mean (S.E)	1.86 (0.04)	1.88 (0.04)	1.78 (0.07)	*t* = 1.18	0.245
Vitamin B_12_ (mcg), mean (S.E)	5.32 (0.21)	5.43 (0.26)	4.91 (0.25)	*t* = 1.37	0.178
Folate (mcg), mean (S.E)	505.71 (11.03)	505.42 (11.68)	506.79 (23.73)	*t* = −0.05	0.957
C-reactive protein (mg/dL), mean (S.E)	0.55 (0.03)	0.55 (0.03)	0.57 (0.04)	*t* = −0.61	0.545
4-PA (nmol/L), mean (S.E)	109.24 (15.10)	114.14 (16.92)	90.50 (15.29)	*t* = 1.37	0.177
PLP (nmol/L), mean (S.E)	57.39 (2.18)	59.46 (2.56)	49.45 (2.27)	*t* = 3.28	0.002
4-PA/PLP, mean (S.E)	1.65 (0.13)	1.54 (0.14)	2.04 (0.27)	*t* = −1.71	0.094
4-PA/PLP, *n* (%)				χ^2^ = 10.645	0.001
<0.89	854 (49.91)	712 (52.48)	142 (40.08)		
≥0.89	844 (50.09)	624 (47.52)	220 (59.92)		
Creatinine (mg/dL), mean (S.E)	1.01 (0.02)	0.97 (0.02)	1.18 (0.05)	*t* = −4.23	<0.001
LDH (U/L), mean (S.E)	136.32 (0.87)	134.74 (0.99)	142.41 (2.21)	*t* = −3.04	0.004
Platelet (1,000 cells/uL), mean (S.E)	258.48 (2.90)	259.67 (3.28)	253.94 (4.81)	*t* = 1.05	0.301
EASIX, mean (S.E)	0.63 (0.02)	0.59 (0.02)	0.80 (0.05)	*t* = −3.78	<0.001
Log EASIX, mean (S.E)	−0.98 (0.03)	−1.04 (0.04)	−0.77 (0.06)	*t* = −3.93	<0.001
Log EASIX, *n* (%)				χ^2^ = 9.705	0.002
<−0.73	1,069 (66.99)	873 (69.46)	196 (57.51)		
≥ − 0.73	629 (33.01)	463 (30.54)	166 (42.49)		

PIR, family poverty-to-income ratio; MET, metabolic equivalent task; CVD, cardiovascular disease; BMI: body mass index; 4-PA, 4-pyridoxine; PLP, pyridoxal 5′-phosphate; LDH, lactate dehydrogenase; EASIX, Endothelial Activation and Stress Index; χ^2^ = chi-square test.

### Association between 4-PA/PLP and log EASIX

We used the weighted univariate logistic regression analysis to screen the covariates, and the result is shown in Supplementary Table S2. Table 2 presents the association between 4-PA/PLP and log EASIX using the weighted logistic regression models. In the unadjusted model, a high level of 4-PA/PLP was associated with an increased level of log EASIX (Model 1: OR = 2.75, 95%CI: 2.19–3.45, *p* < 0.001). After adjusting for age, gender, ethnicity, education level, PIR, physical activity, duration of diabetes, and CVD, the association between 4-PA/PLP and log EASIX remained (Model 2: OR = 1.56, 95%CI: 1.06–2.30, *p* = 0.024).

**Table 2:** Association between 4-PA/ PLP and log EASIX.

Variables	Model 1		Model 2	
	OR (95%CI)	*P*	OR (95%CI)	*P*
4-PA/PLP				
Low level	Ref		Ref	
High level	2.75 (2.19–3.45)	<0.001	1.56 (1.06–2.30)	0.024

4-PA/ PLP: 4-pyridoxine/pyridoxal 5′-phosphate; Ref: reference; OR: odds ratio; CI: confidence interval. Model 1 was the crude model. Model 2 was adjusted for age, gender, ethnicity, education level, family poverty-to-income ratio, physical activity, duration of diabetes, and cardiovascular disease (association between 4-PA/ PLP and log EASIX).

### The mediating effect of log EASIX among the total population and its subgroups

**Figure 2:**
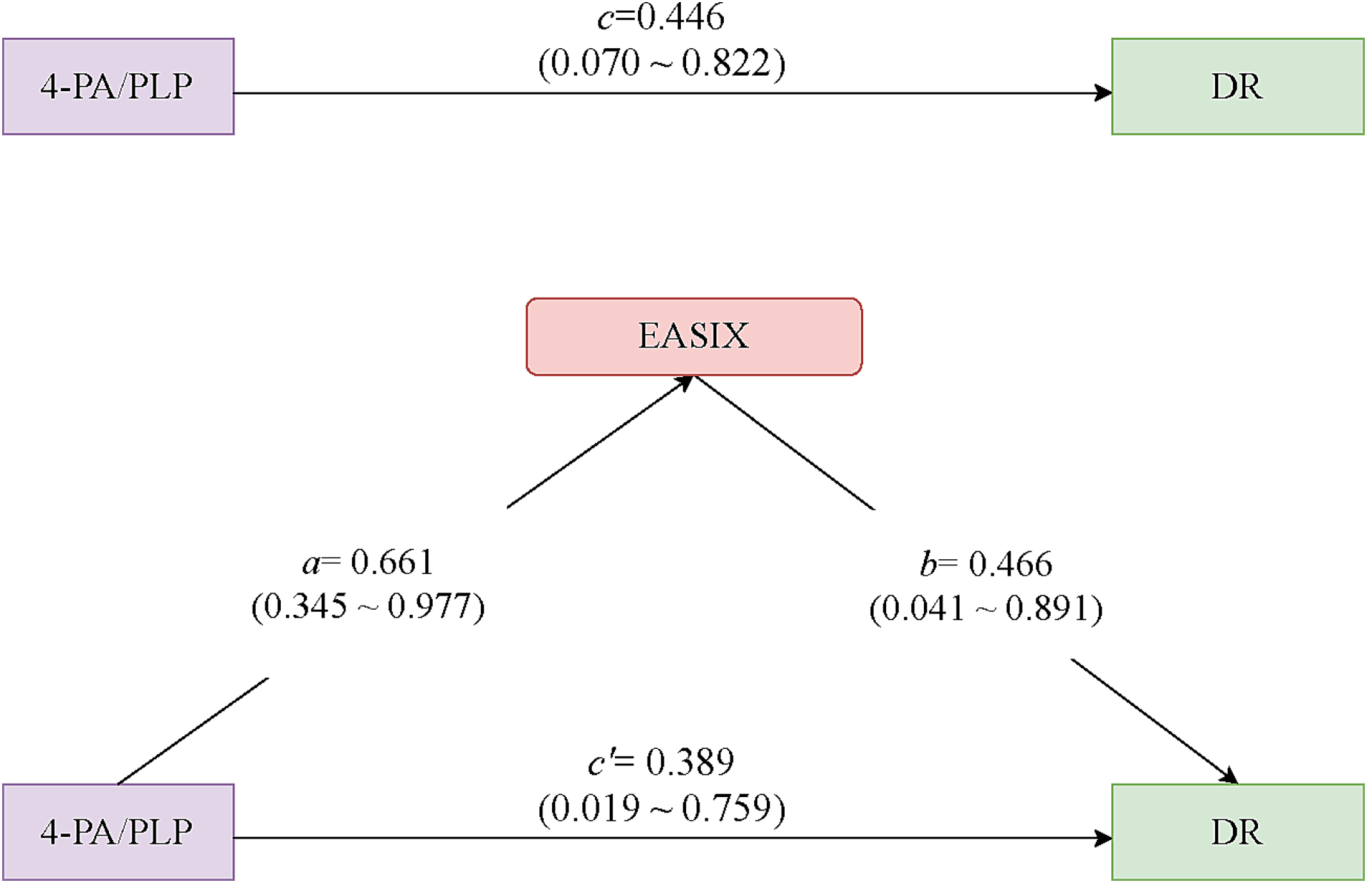
Path diagram of the mediation analysis models. Path a: estimated coefficient for the regression with 4-PA/PLP predicting log EASIX; Path b: estimated coefficient for the regression with log EASIX predicting DR; Path c = total effects for the regression with 4-PA/PLP predicting DR; Path c’ = direct effects for the regression with 4-PA/PLP predicting DR independent of log EASIX. 4-PA/PLP: the ratio of 4-pyridoxine (4-PA) to pyridoxal 5′-phosphate (PLP); EASIX: Endothelial Activation and Stress Index; DR: diabetic retinopathy.

After adjusting for all covariates, a high level of 4-PA/PLP was associated with an increased odd of DR compared to the reference group with low levels of 4-PA/PLP (Model 3A: OR = 1.94, 95%CI: 1.40–2.67, *p* < 0.001). The mediating effect of log EASIX on the association between 4-PA/PLP and DR is presented in Table 3. We found that log EASIX may play a mediating role in the 4-PA/PLP and DR, with a 95% CI of distribution of product of 0.31 (95% CI: 0.02–0.67). The proportion of mediation was 69.06%. Figure 2 shows the path diagram of the mediation analysis models. In addition, the mediating effect of log EASIX was also observed in individuals with diabetes who were aged ≥60 years (proportion of mediation: 50.63%) or had a duration of diabetes ≥10 years (proportion of mediation: 71.83%) (Table 4 and Supplementary Figure S1).

**Table 3:** Mediating effect of log EASIX.

Variables	Model 3A		Model 3B		Distribution of product *β* (95%CI)	Indirect effect OR (95%CI)	Proportion of mediation
	OR (95%CI)	*P*	OR (95%CI)	*P*			
4-PA/PLP					0.31 (0.02–0.67)	1.36 (1.02–1.96)	69.06%
Low level	Ref		Ref				
High level	1.94 (1.40–2.67)	<0.001	1.48 (1.01–2.16)	0.045			
Log EASIX							
Low	/	/	Ref				
High	/	/	1.59 (1.03–2.47)	0.037			

4-PA/ PLP: 4-pyridoxine/pyridoxal 5′-phosphate; Ref: reference; OR: odds ratio; CI: confidence interval. Model 3A was adjusted for age, gender, ethnicity, education level, family poverty-to-income ratio, physical activity, duration of diabetes, and cardiovascular disease (association between 4-PA/ PLP and DR; total effect). Model 3B was adjusted for age, gender, ethnicity, education level, family poverty-to-income ratio, physical activity, duration of diabetes, cardiovascular disease, and log EASIX (direct effect).

**Table 4:** Subgroup analysis.

Subgroup	Variables	Model 4A		Model 4B		Model 4C		Distribution of product *β* (95%CI)	Indirect effect OR (95%CI)	Proportion of mediation
		OR (95%CI)	*P*	OR (95%CI)	*P*	OR (95%CI)	*P*			
Age: <60 years	4-PA/PLP							/	/	/
	Low level	Ref		Ref		Ref				
	High level	1.28 (0.68–2.38)	0.435	1.99 (1.03–3.83)	0.040	1.22 (0.67–2.23)	0.506			
	Log EASIX									
	Low	/	/	/	/	Ref				
	High	/	/	/	/	1.49 (0.79–2.82)	0.213			
Age: ≥60 years	4-PA/PLP							0.35 (0.01–0.75)	1.41 (1.01–2.11)	50.63%
	Low level	Ref		Ref		Ref				
	High level	1.98 (1.11–3.51)	0.021	1.97 (1.50–2.60)	<0.001	1.84 (1.02–3.31)	0.042			
	Log EASIX									
	Low	/	/	/	/	Ref				
	High	/	/	/	/	1.66 (1.01–2.74)	0.048			
Duration of diabetes: <10	4-PA/PLP							/	/	/
	Low level	Ref		Ref		Ref				
	High level	1.28 (0.67–2.42)	0.446	1.87 (1.16–3.03)	0.012	1.26 (0.67–2.36)	0.466			
	Log EASIX									
	Low	/	/	/	/	Ref				
	High	/	/	/	/	1.13 (0.55–2.32)	0.725			
Duration of diabetes: ≥10	4-PA/PLP							0.49 (0.03–1.18)	1.64 (1.03–3.24)	71.83%
	Low level	Ref		Ref		Ref				
	High level	1.98 (1.33–2.96)	0.001	2.00 (1.18–3.41)	0.012	1.84 (1.22–2.76)	0.004			
	Log EASIX									
	Low	/	/	/	/	Ref				
	High	/	/	/	/	2.03 (1.09–3.78)	0.026			

4-PA/ PLP: 4-pyridoxine/pyridoxal 5′-phosphate; Ref: reference; OR: odds ratio; CI: confidence interva. Model 4A was adjusted for age, gender, ethnicity, education level, family poverty-to-income ratio, physical activity, duration of diabetes, and cardiovascular disease (association between 4-PA/PLP and log EASIX). Model 4B was adjusted for age, gender, ethnicity, education level, family poverty-to-income ratio, physical activity, duration of diabetes, and cardiovascular disease (association between 4-PA/PLP and DR; total effect). Model 4C was adjusted for age, gender, ethnicity, education level, family poverty-to-income ratio, physical activity, duration of diabetes, cardiovascular disease and log EASIX (direct effect). The adjustment of variables is not performed when they serve as subgroup variables.

## Discussion

In the present study, we explored the associations between 4-PA/PLP, EASIX, and DR among patients with diabetes using the NHANES data. The findings suggested positive associations between high levels of 4-PA/PLP and log EASIX, as well as between high levels of 4-PA/PLP and DR. Interestingly, we found that log EASIX mediates the association between 4-PA/PLP and DR, with a mediation proportion of 69.06%. A significant mediating effect of log EASIX was more likely to occur among individuals with diabetes who were aged ≥60 years or had a duration of diabetes ≥10 years.

Vitamin B_6_ is a crucial nutrient. There was evidence linking the relationship between vitamin B_6_ and DR risk. An analysis of the data from the Japan Diabetes Complications Study revealed a significant inverse association between high vitamin B6 intake and the incidence of DR in individuals with type 2 diabetes in Japan, suggesting a potential protective role of increased vitamin B_6_ consumption (10). In a narrative review, a high dietary intake of vitamin B_6_ was also found to significantly reduce the risk of DR following an 8-year follow-up period (17). The involvement of inflammation in the pathogenesis of DR is widely acknowledged (18). Vitamin B_6_ has been recognized for its antioxidant and anti-inflammatory properties, as well as its ability to modulate immunity and gene expression (19). An anti-inflammatory diet intake may decrease the levels of inflammatory markers and consequently lower the likelihood of developing DR. In addition, the deficiency of vitamin B_6_ leads to an increase in homocysteine levels (20). Elevated homocysteine is considered a risk factor for DR (21). Similar to previous research, our study found that a high level of 4-PA/PLP was related to increased DR risk. PLP serves as a coenzyme form of vitamin B_6_, playing a pivotal role in catalyzing over 160 diverse functions (22). 4-PA represents the primary metabolite of vitamin B_6_ (23). 4-PA/PLP was used as an indicator for assessing vitamin B_6_ turnover, reflecting a state of low vitamin B_6_ status due to altered tissue distribution or increased vitamin B_6_ turnover (24).

Endothelial cells play crucial roles in maintaining retinal vascular homeostasis (25). Retinal vascular dysfunction is induced by various factors, including advanced glycosylation end products and receptors, oxidative stress, pro-inflammatory cytokines and chemokines, proliferator-activated receptor-*γ* disruption, growth factors, and microRNA; these factors contribute to the impairment of retinal endothelial function, ultimately leading to the development of DR (4). The focus on markers of endothelial dysfunction as biomarkers of DR may be reasonable. EASIX, as a marker of endothelial injury, has been validated as an easily predictive biomarker for the prediction of survival outcomes in various medical conditions (26–28). However, there is a lack of research investigating the correlation between EASIX and the progression of DR. In this study, we observed that a high level of log EASIX was associated with an increased risk of DR. In addition, a positive association between 4-PA/PLP and log EASIX was found in this study. Oxidative stress is characterized by the imbalance between oxidation product levels and the body’s antioxidant capacity (29). Several studies revealed that nutrition rich in antioxidant properties possesses the ability to effectively scavenge free radicals and reactive oxygen species (ROS), thereby mitigating oxidative stress among individuals with diabetes (30, 31). A higher level of vitamin B_6_ was found to normalize endothelial dysfunction in children with type 1 diabetes (32). This might explain the relationship between 4-PA/PLP and log EASIX.

To the best of our knowledge, this is the first study to explore the mediating effect of log EASIX for the association between 4-PA/PLP and DR among individuals with diabetes based on the NHANES data. The results showed that log EASIX partially mediated the relationship between 4-PA/PLP and DR risk, offering valuable insights into the potential role of endothelial function indicators in this relationship. In addition, the mediation proportion of log EASIX was 69.06%, indicating that it was largely involved in the process of 4-PA/PLP on the development of DR. In other words, the association between 4-PA/PLP and DR risk was serially mediated through the 4-PA/PLP-endothelial dysfunction path and the endothelial dysfunction-DR path, suggesting that 4-PA/PLP accelerated endothelium dysfunction and finally aggravated the DR progress.

The finding emphasizes the importance of reducing DR risk by improving endothelial dysfunction, especially in populations with high 4-PA/PLP. Simultaneously, we observed the specificity of age and diabetes duration in the mediating role of log EASIX between 4-PA/PLP and DR risk using stratified analysis. Log EASIX was not mediated in the individuals with diabetes who were aged <60 years, and it might be suggested that for the individuals with diabetes who were aged ≥60 years, reducing the EASIX would be the focus for preventing DR occurrence. Furthermore, among individuals with diabetes and a duration of diabetes ≥10 years, log EASIX partially mediated the association between 4-PA/PLP and DR risk (mediation proportion: 71.83%). However, among those with a duration of diabetes <10 years, the effect of 4-PA/PLP on the development of DR did not appear to be mediated by log EASIX. This finding may provide new insights into the reduced risk of DR in diabetes duration-specific populations.

Nevertheless, some limitations of our study should not be ignored. First, our study was cross-sectional; we cannot establish a causal relationship between 4-PA/PLP, EASIX, and DR. Second, we adjusted for many potential confounders, but we cannot entirely exclude the possibility of unmeasured confounders, such as medication therapy. Third, data on DR were collected by self-report, which might potentially introduce recall bias. In addition, due to the substantial amount of missing or unavailable data on vitamin B6, vitamin B12, and folate supplements in the NHANES database, the data collected for this study solely relied on dietary intake. Finally, similar to the majority of NHANES studies, the results of this study are limited to the US population, and the results need to be interpreted with caution. Further research is essential to confirm our findings in a prospective study.

## Conclusion

This study identified a positive association between high levels of 4-PA/PLP and an increased risk of DR, with the relationship partially mediated by log EASIX. Further prospective studies are necessary to examine the effects of EASIX on the relationship between 4-PA/PLP and DR.

## Data Availability

Publicly available datasets were analyzed in this study. This data can be found here: NHANES database, https://wwwn.cdc.gov/nchs/nhanes/.

## Supplementary material

SUPPLEMENTARY FIGURE S1 The mediating effect of log EASIX on the association between 4-PA/PLP and DR risk in the age and diabetes duration subgroups. 4-PA/PLP: the ratio of 4-pyridoxine (4-PA) to pyridoxal 5’-phosphate (PLP); EASIX: Endothelial Activation and Stress Index; DR: diabetic retinopathy.
