# Supplementary figures and images for: Mediation of endothelial activation and stress index in the association between vitamin B6 turnover rate and diabetic retinopathy: an analysis of the National Health and Nutrition Examination Survey
Source: Front Nutr. 2025 Jan 14;11:1490340. doi: 10.3389/fnut.2024.1490340 (PMC11772182; doi:10.3389/fnut.2024.1490340)

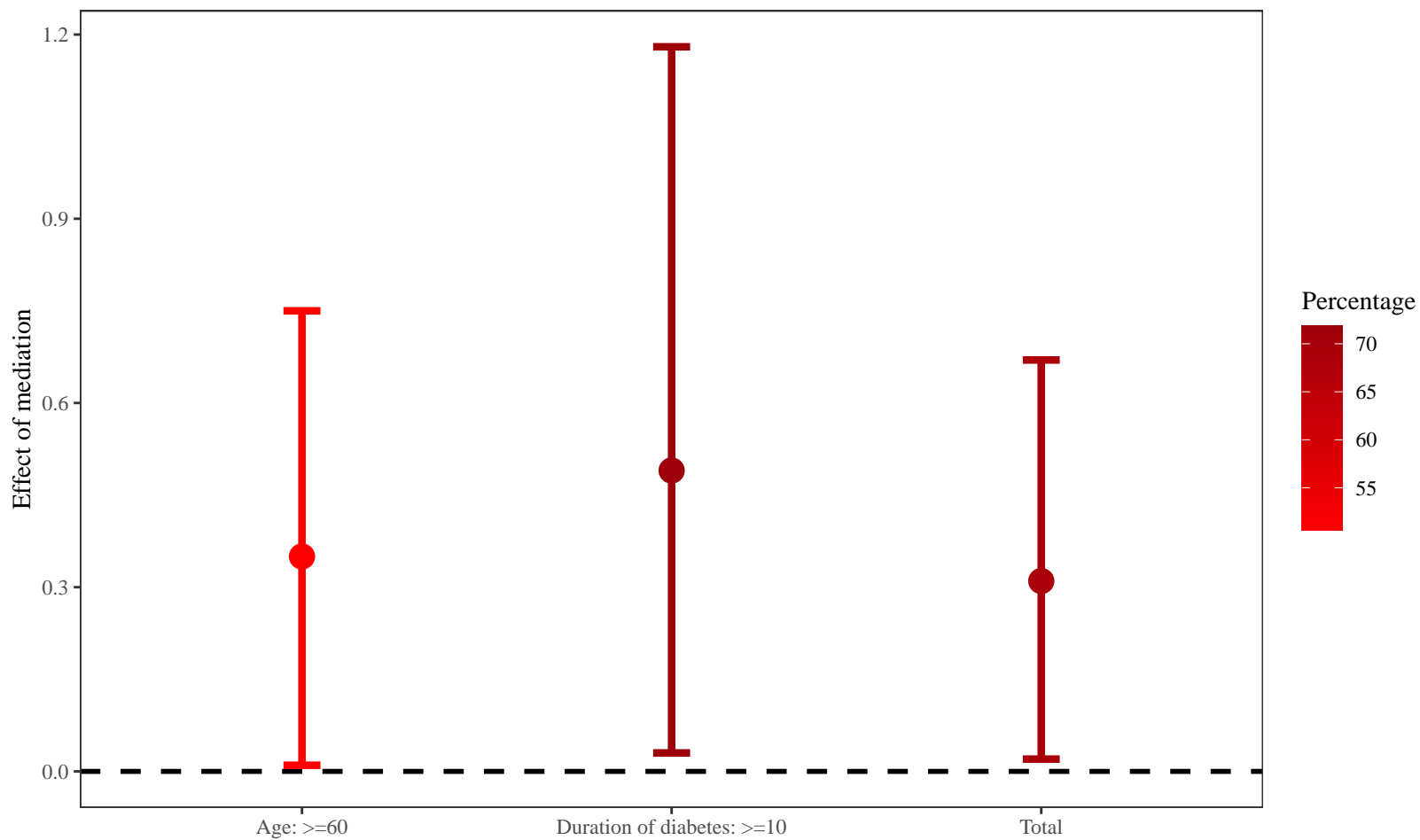

Supplement: SUPPLEMENTARY FIGURE S1 — The mediating effect of log EASIX on the association between 4-PA/PLP and DR risk in the age and diabetes duration subgroups. 4-PA/PLP: the ratio of 4-pyridoxine (4-PA) to pyridoxal 5’-phosphate (PLP); EASIX: Endothelial Activation and Stress Index; DR: diabetic retinopathy. [file Image_1.pdf]
