# Supplementary material for: Mediation of endothelial activation and stress index in the association between vitamin B6 turnover rate and diabetic retinopathy: an analysis of the National Health and Nutrition Examination Survey
Source: Front Nutr. 2025 Jan 14;11:1490340. doi: 10.3389/fnut.2024.1490340 (PMC11772182; doi:10.3389/fnut.2024.1490340)
Supplement: Supplementary file 2 [file Table_1.docx]

**Supplementary Table 1** **Sensitivity analysis**

| Variables | After interpolation | Before interpolation | Statistics | *P* |
| --- | --- | --- | --- | --- |
| PIR, Mean (S.E) | 2.69 (0.05) | 2.71 (0.06) | t=-1.23 | 0.225 |
| BMI (kg/m^2^), Mean (S.E) | 32.77 (0.26) | 32.78 (0.26) | t=-0.23 | 0.818 |
| Energy (kcal), Mean (S.E) | 1870.54 (29.11) | 1876.67 (30.16) | t=-1.25 | 0.216 |
| Vitamin B_6_ (mg), Mean (S.E) | 1.86 (0.04) | 1.86 (0.04) | t=-0.08 | 0.938 |
| Vitamin B_12_ (mcg), Mean (S.E) | 5.32 (0.21) | 5.34 (0.22) | t=-0.69 | 0.492 |
| Folate, (mcg), Mean (S.E) | 505.71 (11.03) | 505.47 (11.35) | t=0.11 | 0.910 |
| Education level, n (%) |  |  | χ^2^=2.364 | 0.307 |
| Below high school | 699 (29.60) | 697 (29.59) |  |  |
| High school | 378 (24.22) | 378 (24.23) |  |  |
| Above high school | 621 (46.18) | 620 (46.18) |  |  |
| Duration of diabetes (years), Mean (S.E) | 10.84 (0.30) | 10.77 (0.31) | t=1.53 | 0.134 |

PIR: family poverty to income ratio; BMI: body mass index; χ^2^= Chi-square test.

**Supplementary Table 2 Screening of covariates by weighted univariate logistic regression analysis**

| Variables | OR (95%CI) | *P* |
| --- | --- | --- |
| Age | 1.00 (0.99-1.01) | 0.927 |
| Gender |  |  |
| Male | Ref |  |
| Female | 1.20 (0.88-1.64) | 0.253 |
| Race |  |  |
| White | Ref |  |
| Black | 1.26 (0.94-1.70) | 0.125 |
| Others | 1.07 (0.75-1.53) | 0.686 |
| Education level |  |  |
| Below high school | Ref |  |
| High school | 0.86 (0.62-1.20) | 0.376 |
| Above high school | 0.83 (0.55-1.23) | 0.343 |
| PIR |  |  |
| <1 | Ref |  |
| ≥1 | 0.59 (0.44-0.79) | <0.001 |
| Smoking status |  |  |
| No | Ref |  |
| Yes | 0.92 (0.63-1.35) | 0.662 |
| Physical activity |  |  |
| <750 MET·min/week | Ref |  |
| ≥750 MET·min/week | 0.71 (0.52-0.97) | 0.033 |
| Unknown | 1.06 (0.69-1.64) | 0.775 |
| Duration of diabetes | 1.04 (1.02-1.05) | <0.001 |
| Duration of diabetes |  |  |
| <10 | Ref |  |
| ≥10 | 2.64 (1.88-3.72) | <0.001 |
| Hypertension |  |  |
| No | Ref |  |
| Yes | 1.24 (0.80-1.92) | 0.335 |
| Dyslipidemia |  |  |
| No | Ref |  |
| Yes | 1.48 (0.85-2.57) | 0.164 |
| CVD |  |  |
| No | Ref |  |
| Yes | 1.62 (1.15-2.29) | 0.007 |
| Cancer |  |  |
| No | Ref |  |
| Yes | 0.86 (0.51-1.44) | 0.551 |
| BMI | 1.01 (0.99-1.04) | 0.222 |
| BMI, kg/m^2^ |  |  |
| <25 | Ref |  |
| 25~30 | 1.05 (0.65-1.71) | 0.830 |
| ≥30 | 1.12 (0.71-1.77) | 0.624 |
| Energy | 1.00 (1.00-1.00) | 0.342 |
| Vitamin B_6_ | 0.92 (0.80-1.07) | 0.270 |
| Vitamin B_12_ | 0.99 (0.96-1.01) | 0.288 |
| Folate | 1.00 (1.00-1.00) | 0.956 |
| C-reactive protein | 1.05 (0.91-1.21) | 0.540 |

PIR: family poverty to income ratio; MET: metabolic equivalent task; CVD: cardiovascular disease; BMI: body mass index; OR: odds ratio; CI: confidence interval.
